# Supplementary material for: Environment-induced heritable variations are common in Arabidopsis thaliana
Source: Nat Commun. 2024 May 30;15:4615. doi: 10.1038/s41467-024-49024-3 (PMC11139905; doi:10.1038/s41467-024-49024-3)
Supplement: Supplementary file 1 — Supplementary Information [file 41467_2024_49024_MOESM1_ESM.pdf]

Supplementary Information

Environment-induced heritable variations are common in *Arabidopsis thaliana*

Xiaohe Lin[1†], Junjie Yin[1†], Yifan Wang[1†], Jing Yao[1], Qingshun Q. Li[1,2], Vit Latzel[3], Oliver Bossdorf[4], Yuan-Ye Zhang[1*]

[1]Key Laboratory of the Ministry of Education for Coastal and Wetland Ecosystems, College of the Environment and Ecology, Xiamen University, Xiamen, Fujian, China

[2]Biomedical Sciences, College of Dental Medicine, Western University of Health Sciences, Pomona, California, USA

[3]Institute of Botany of the CAS, Zamek 1, 252 43 Pruhonice, Czech Republic

[4]Institute of Evolution & Ecology, University of Tübingen, Auf der Morgenstelle 5, 72076 Tübingen, Germany

[†] These authors contributed equally: Xiaohe Lin, Junjie Yin, Yifan Wang

[*] Correspondence: zhangyuanye@xmu.edu.cn (Y-Y.Z.)

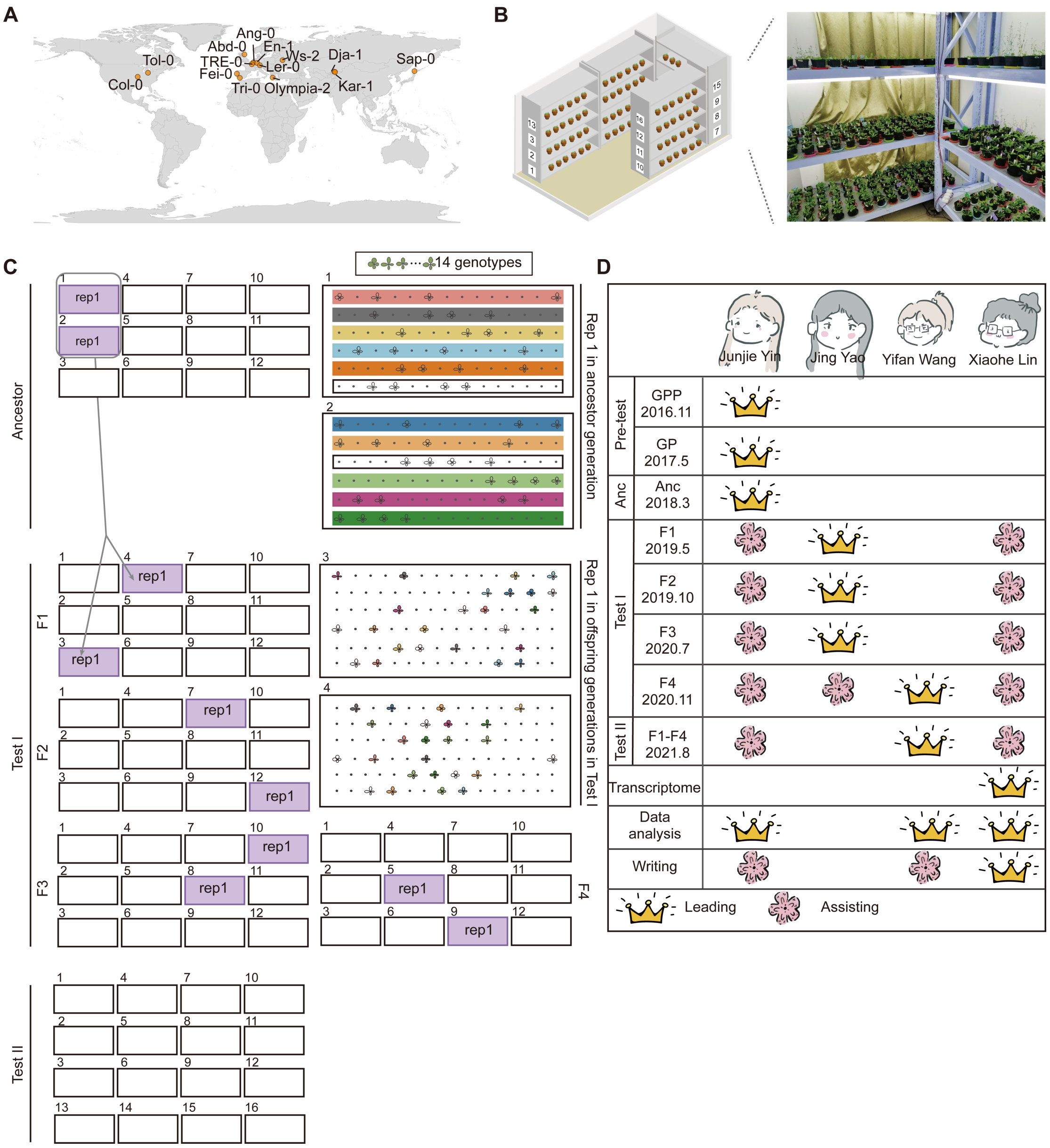

Supplementary Fig. 1| Details of the experimental design. **a**, The geographic origins of the 14 accessions of *A. thaliana*. **b**, The growth room setup and numbers indicating different layers. **c**, The design and randomization of the experiment. Colour shades indicate different treatments. **d,** The timeline of this study and the contributions of student authors.

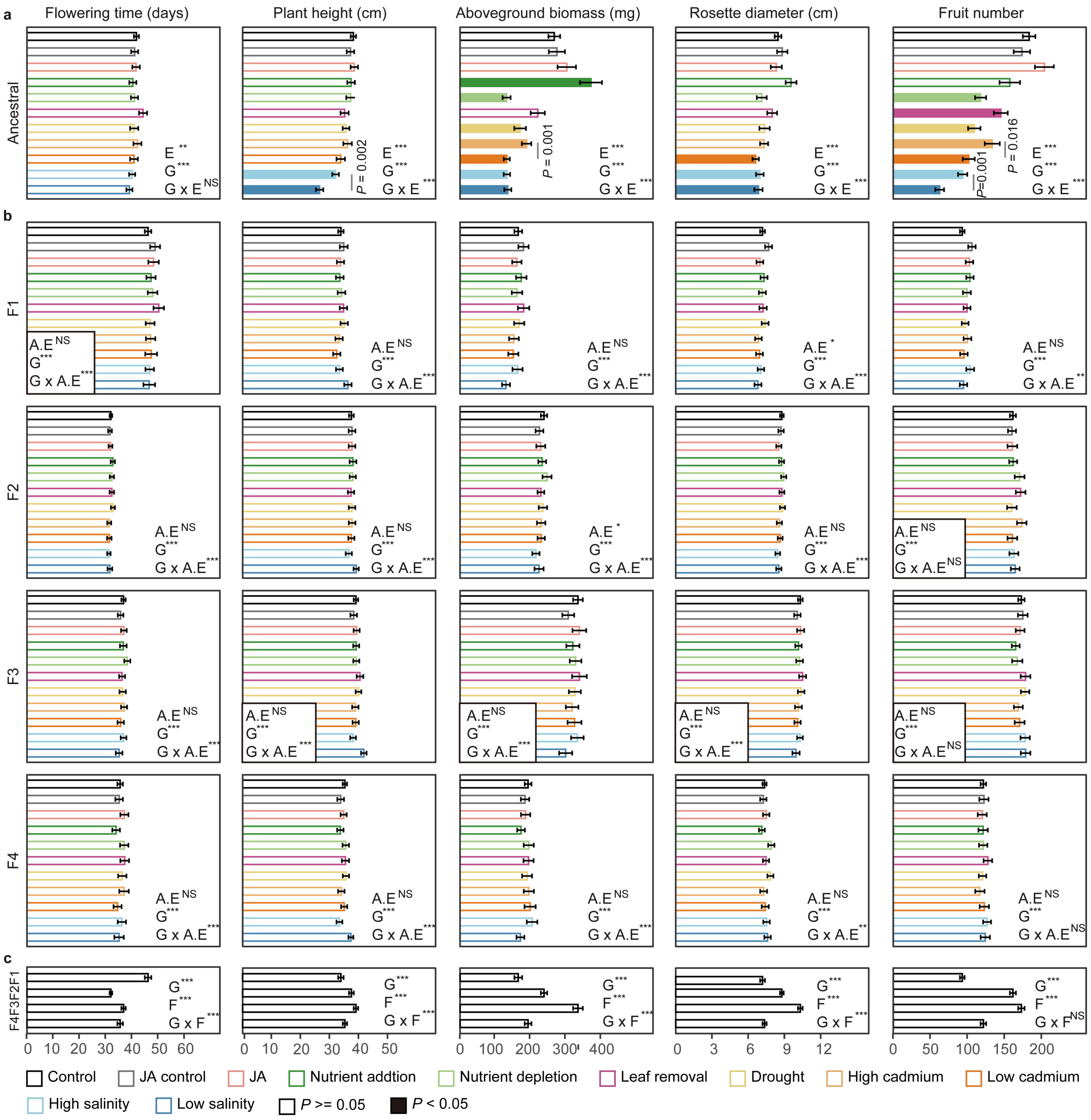

Supplementary Fig. 2| Phenotypes of the ancestral generation and offspring generations (Test I). a, Phenotypes (mean ± SE) of the ancestral generation. Filled bars indicate that the treatment significantly changed the phenotype compared to the control environment. The *P* values for multiple comparisons are adjusted with the "single-step" method in the function *glht* (R package "multcomp"). **b**, The phenotypes (mean ± SE) of offspring generations (F1-F4). All phenotypes were measured in the control environment in Test I. **c**, A comparison of phenotypes (mean ± SE) between offspring generations of plants derived from the ancestral environment of the control. The means are estimated from six independent biological replicates (n = 6) per genotype/treatment/generation. E, A.E, G, and F represent fixed effects of the environment, ancestral environment, genotype and generation. Significance levels: *, $P < 0.05$; **, $P < 0.01$; ***, $P < 0.001$. Details of the statistics and *P* values of panels a, b and c are shown in Supplementary Tables 2 and 3. Source data are provided as a Source Data file.

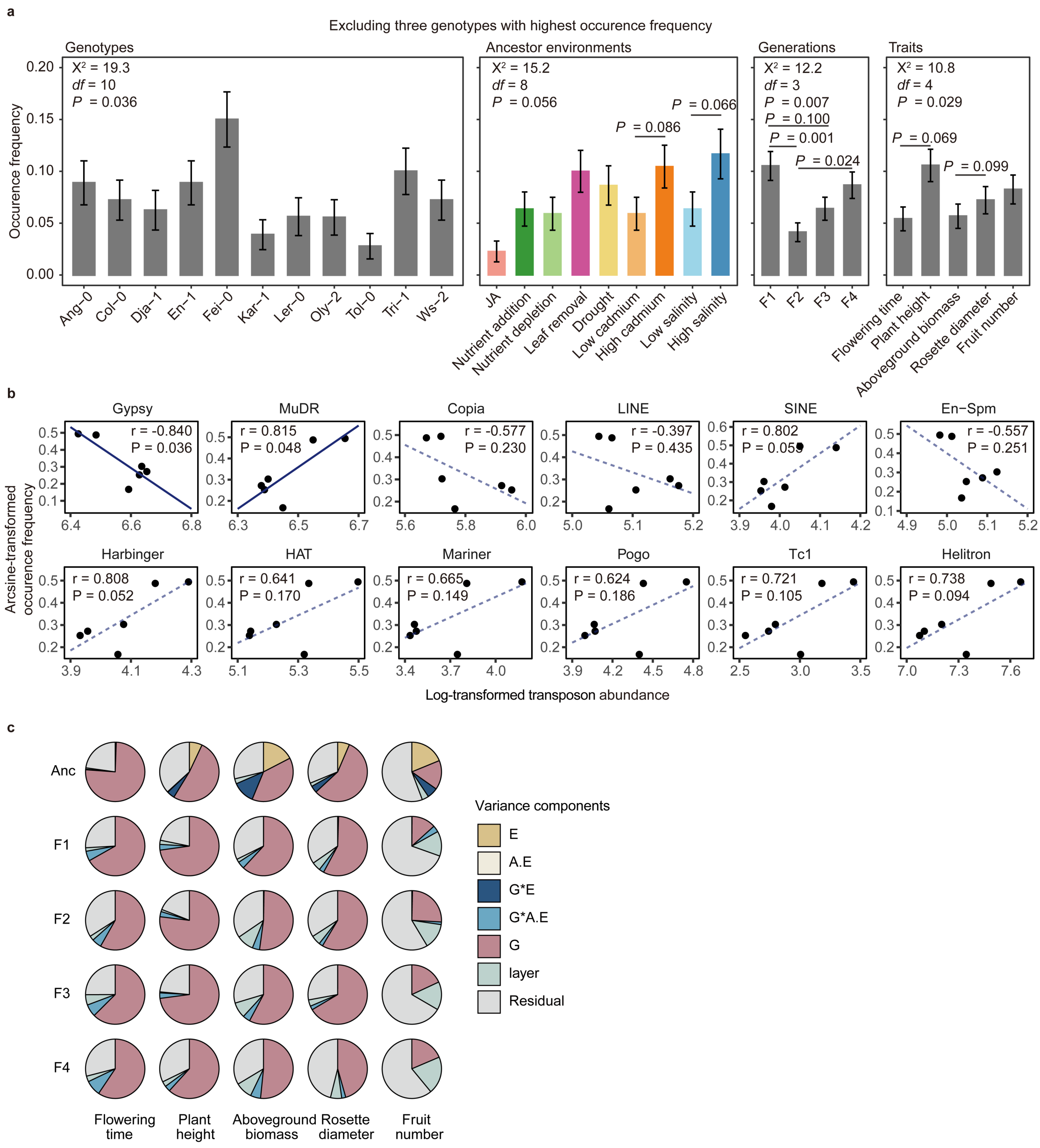

Supplementary Fig. 3 | Predictability of environment-induced phenotypic changes. a, The occurrence frequency (mean ± SE) of significant environment-induced changes. After excluding the three genotypes with the highest occurrence frequencies, the variation between ancestral environmental treatments became insignificant. The occurrence frequencies are estimated from six independent biological replicates (n = 6) per genotype/treatment/ generation. The *P* values for multiple comparisons are adjusted with the "single-step" method in the function *glht* (R package “multcomp”). **b,** The arcsine regressions transformed occurrence frequency among various genotypes against factors that significantly explained this variance (Fig. 3b). **c**, The partitioning of variance in the linear mixed-effect models. Source data are provided as a Source Data file.

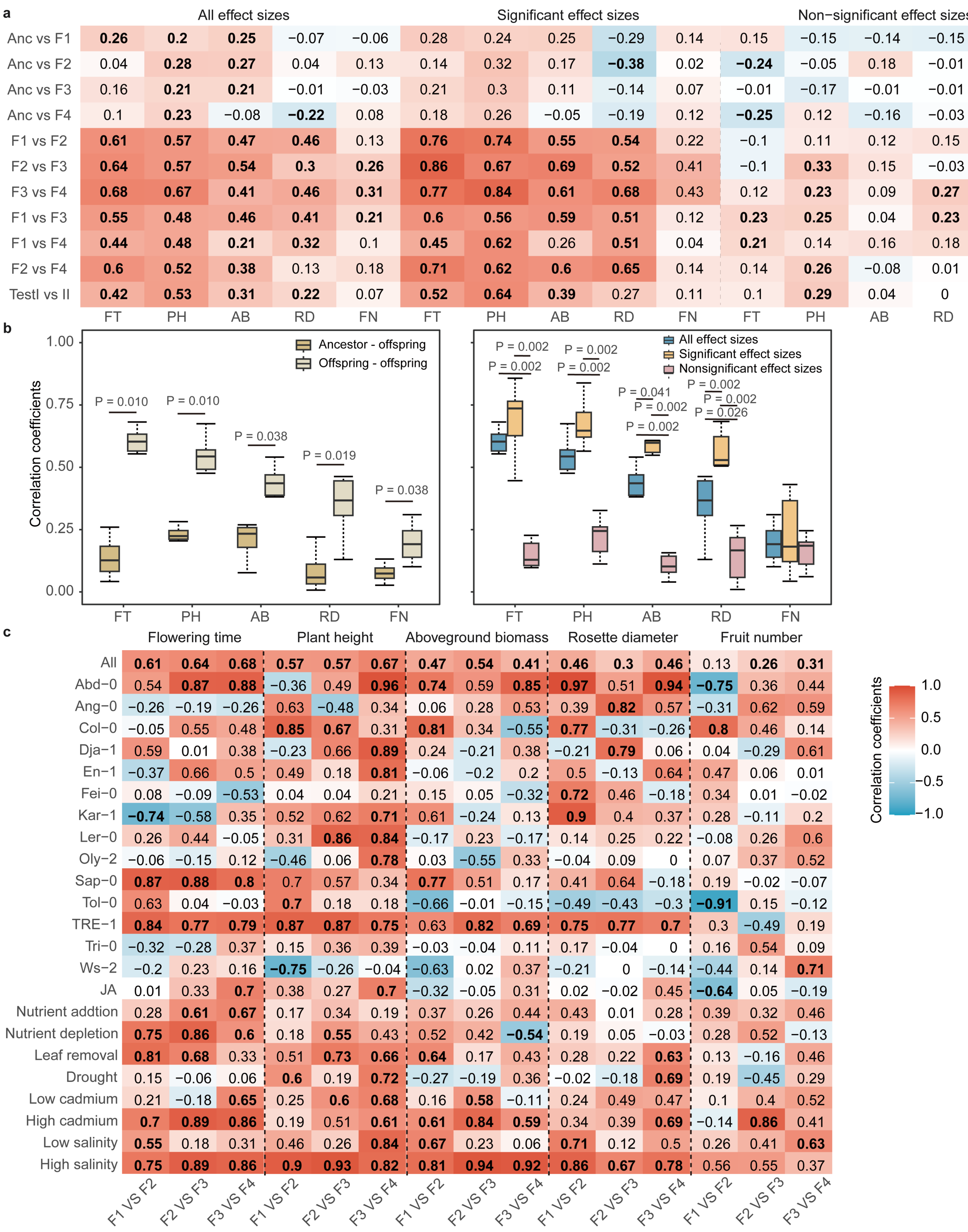

Supplementary Fig. 4 | Reproducibility of effect sizes between generations and tests a, The correlation coefficients between ancestral responses and effect sizes of environment-induced changes in offspring generations**,** between offspring generations, and between tests. Colour scales indicate the correlation coefficients. Significant correlations are shown in bold. **b**, Comparison of correlation coefficients. **c**, The correlation coefficients of effect sizes between offspring generations when we scaled down the experiment to include only one genotype (but multiple treatments) or one treatment (but multiple genotypes). FT: Flowering time, PH: Plant height, AB: Aboveground biomass, RD: Rosette diameter, and FN: Fruit number. Significant correlations are shown in bold. Source data are provided as a Source Data file.

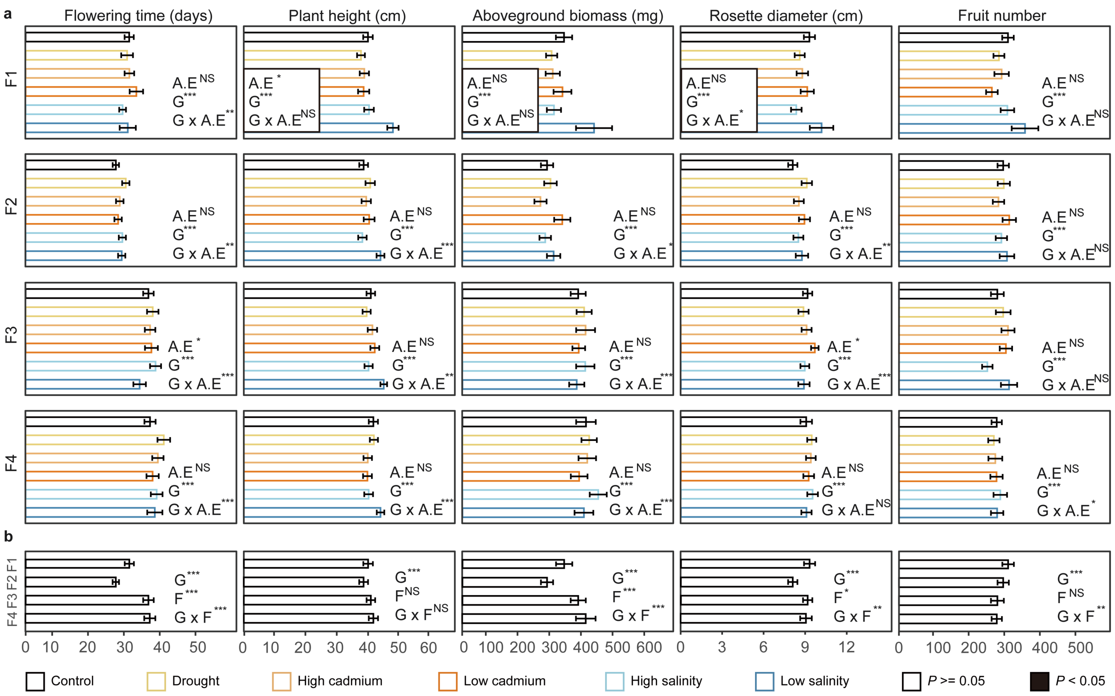

Supplemenary Fig. 5 | The phenotypes of the offspring generation in Test II. a, The phenotypes (mean ± SE) of offspring generations (F1-F4) measured in the control environment. **b,** A comparison of phenotypes (mean ± SE) between generations of plants derived from the ancestral control environment. The estimates are obtained from six independent biological replicates (n = 6) per genotype/treatment/generation. The estimates for F1 may be biased due to missing samples. E, A.E, G, and F represent fixed effects of the environment, ancestral environment, genotype and generation. Significance levels: *, $P < 0.05$; **, $P < 0.01$; ***, $P < 0.001$. Details of the statistics and P values of panels a and b are shown in Supplementary Tables 5 and 6. Source data are provided as a Source Data file.

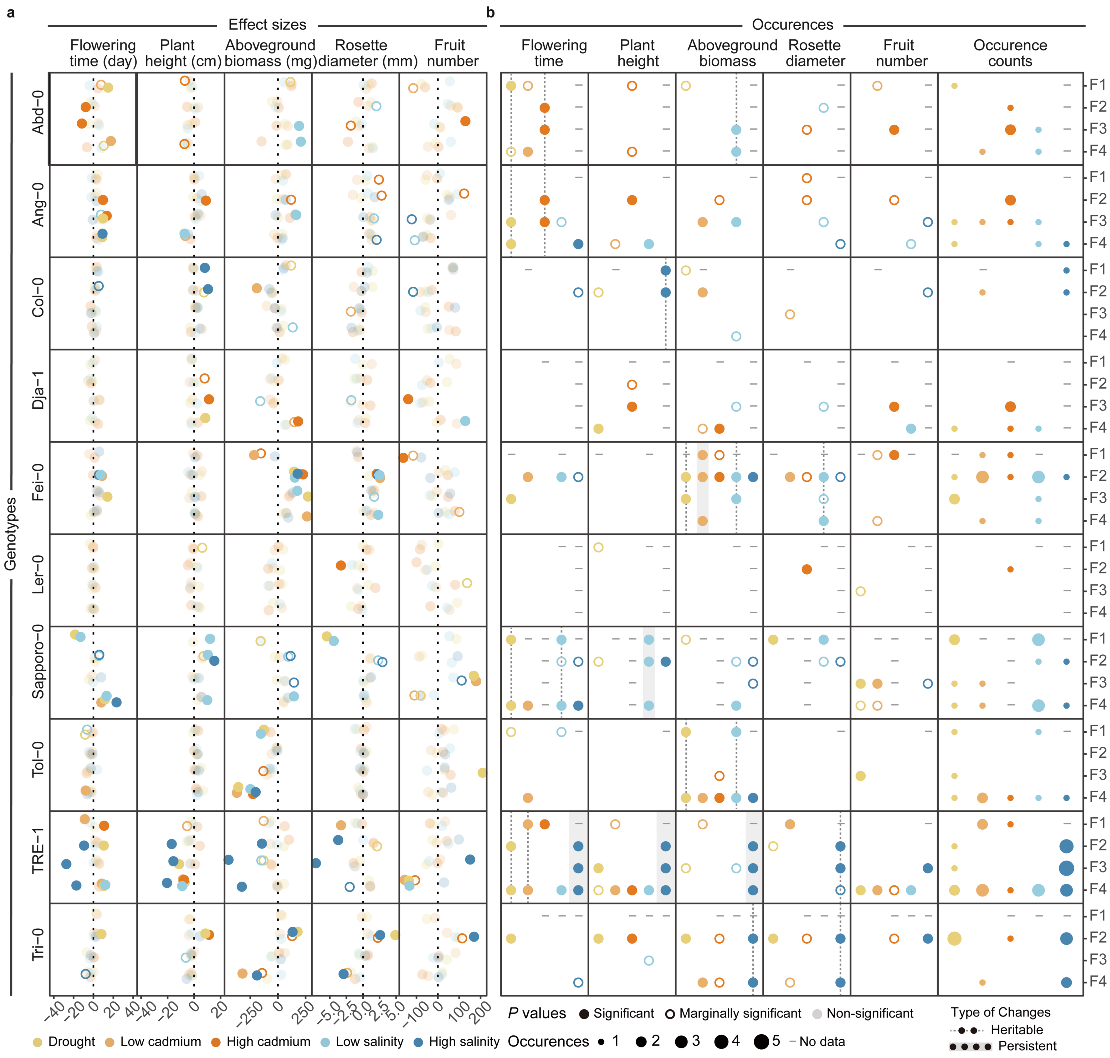

Supplementary Fig. 6 | Ancestral environment-induced phenotypic changes assessed by Test II for different genotypes, environmental treatments and offspring generations. a, The effect sizes of phenotypic changes measured in Test II. **b,** The occurrence of significant and marginally significant effect sizes and the sum of occurrence over phenotypes. Source data including *P* values and statistics are provided as a Source Data file.

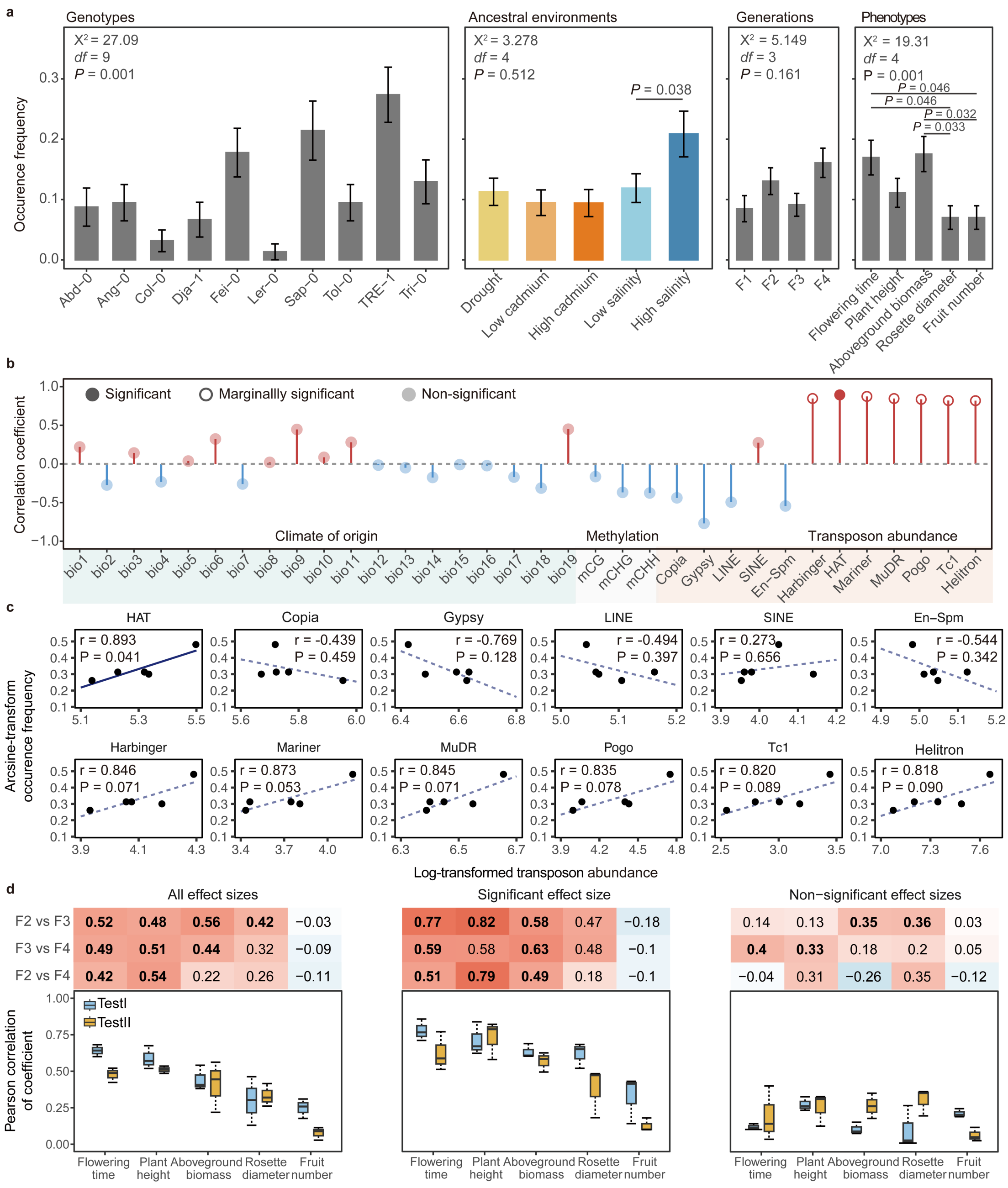

Supplementary Fig. 7 | Predictability and reproducibility analysis of environment-induced changes assessed in Test II. a, The occurrence frequency (mean ± SE) of significant environment-induced changes for different genotypes, environmental treatments, offspring generations and phenotypes. The occurrence frequencies are estimated from six independent biological replicates (n = 6) per genotype/treatment/ generation. The *P* values for multi-comparison were adjusted with the "single-step" method in the function *glht* (R package "multcomp"). b, The correlation coefficients of regressions of occurrence frequency for various genotypes (arcsine transformed) against factors that potentially explain this variance. **c,** The arcsine regressions transformed occurrence frequency of various genotypes against factors that significantly explained this variance. **d,** The correlation coefficients (=reproducibility) of effect sizes between offspring generations and comparisons of reproducibility between Test I and Test II. Source data are provided as a Source Data file.

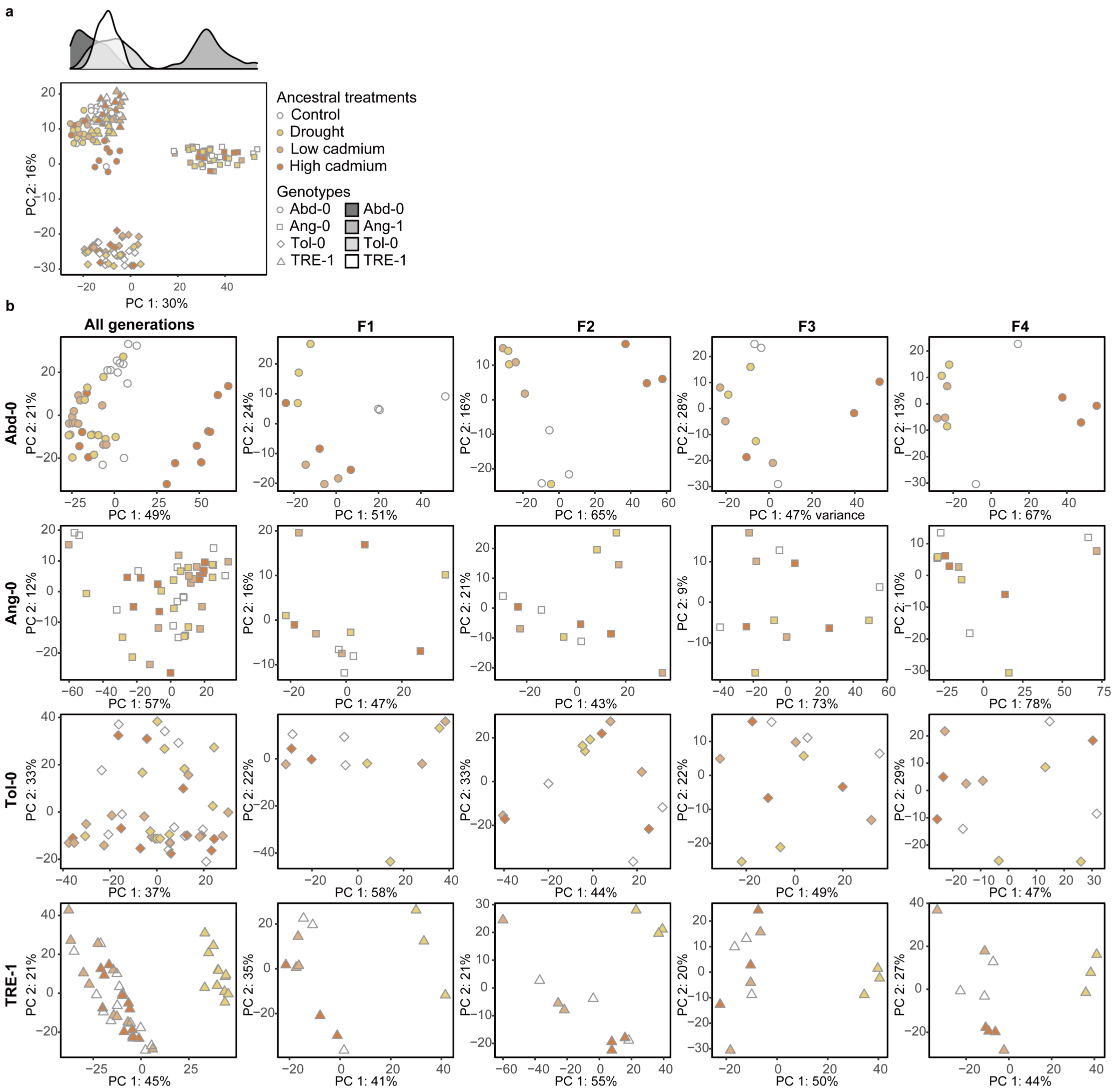

Supplementary Fig. 8 | Principal component analysis (PCA) of genome-wide gene expression patterns of different samples. a, PCA of all samples, including four genotypes, four environmental treatments, and four offspring generations. **b**, PCA for each genotype and generation. Source data are provided as a Source Data file.

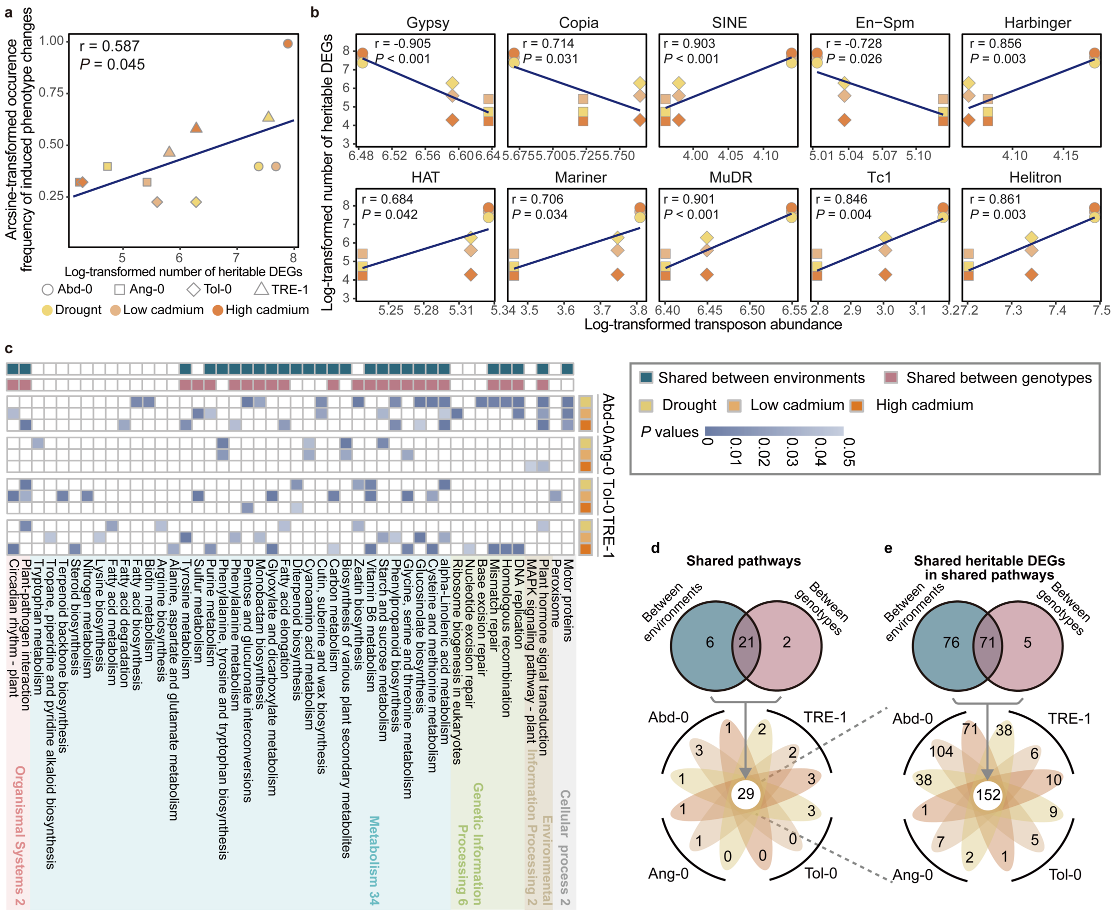

Supplementary Fig. 9: Summary of findings showing the relationships and functions of heritable DEGs. a, The significant positive correlations between occurrence frequencies and numbers of heritable DEGs. **b,** Regressions of the number of heritable DEGs against the number of transposons per superfamily. **c,** The results of KEGG enrichment analysis of heritable DEGs. **d,** Venn plot showing the number of significantly enriched pathways shared between genotypes/treatments. **e,** Venn plot of the number of heritable DEGs within the shared pathways. Source data are provided as a Source Data file.

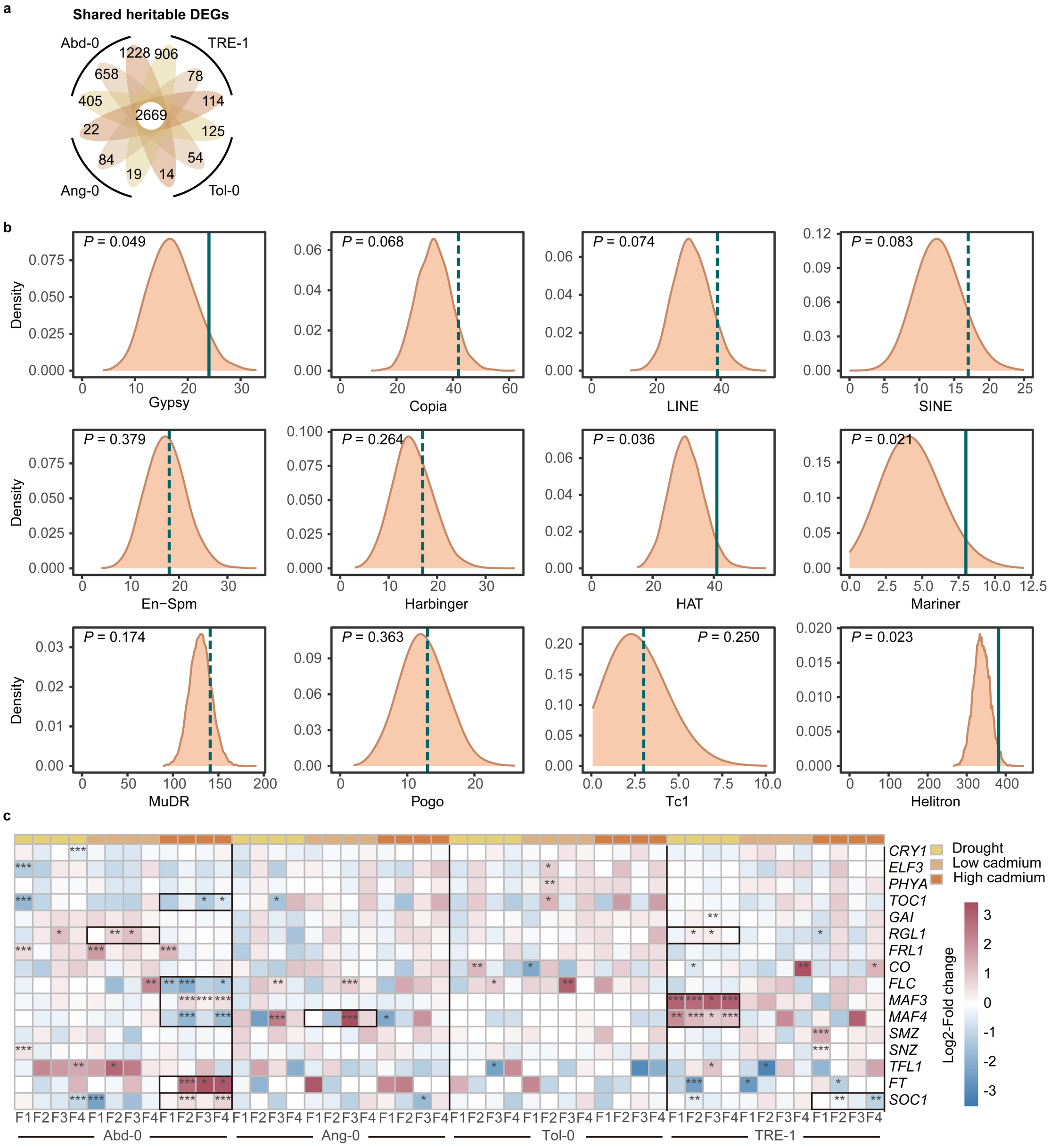

Supplementary Fig. 10 | Shared heritable DEGs and DEGs related to flowering time. a, Venn plot of all heritable DEGs. The number (2669) in the middle circle indicate the number of heritable DEGs that were shared by at least two environments or genotypes. **b**, The null distribution of transposon abundance upstream of genes for different superfamilies. The horizontal lines indicate the observed transposon abundance upstream of the 2669 heritable DEGs shared by at least two environments or genotypes. Significant *P* values indicate significant enrichment of transposons upstream of heritable DEGs compared with the null expectation. **c**. Heatmap of flowering time-related genes that were DEGs for at least one generation. Source data including *P* values and statistics are provided as a Source Data file

Supplementary Table 1 | Geographical, climatic and genomic information of genotypes. This table presents geographical and climatic data sourced from WorldClim (https://worldclim.org), alongside genomic information for the studied genotypes. Geographic information was extracted at a 30-second spatial resolution. Methylation data for 14 genotypes were gathered from published literature (Kawakatsu. et al. Cell, 2016, 166:492-505) and processed to estimate methylation levels across various contexts. The numbers of transposable elements are also extracted from published sources (Quadrana. et al. eLife, 2016, 5:e15716). The original file contained copy number estimation based on read coverage for the 317 TE families analyzed across 211 *A. thaliana* accessions. We summarized the total number of TEs according to superfamilies and then used log-transformed counts for further analysis.

Genotype	Stock number	Longitude	Latitude	Country	State/Province	Bioclim variables																		
						bio1	bio2	bio3	bio4	bio5	bio6	bio7	bio8	bio9	bio10	bio11	bio12	bio13	bio14	bio15	bio16	bio17	bio18	bio19
Abd-0	CS76429	-2.22	57.15	United Kingdom	Aberdeen	76	71	39	4013	175	-6	181	55	43	128	27	812	81	55	15	237	168	197	209
Ang-0	CS76436	5.30	50.30	Belgium	Namur	92	79	34	5632	219	-11	230	163	53	163	20	891	84	62	9	242	194	242	219
Col-0	CS76778	-92.30	38.30	USA	Missouri	130	133	33	9244	324	-76	400	182	4	245	4	1007	124	40	27	323	154	280	154
Dja-1	CS76473	73.62	42.59	Kyrgyzstan	Chui	6	113	27	10234	214	-198	412	69	121	131	-133	511	83	21	44	215	82	121	86
En-1	CS76841	8.50	50.00	Germany	Hessen	97	87	32	6557	243	-22	265	180	25	180	12	606	66	37	18	192	118	192	130
Fei-0	CS76412	-8.54	40.92	Portugal	Aveiro	142	88	44	3850	246	48	198	99	189	190	91	1164	159	14	52	472	79	95	451
Kar-1	CS76522	74.37	42.30	Kyrgyzstan	Chui	9	113	27	10414	217	-201	418	74	-134	134	-134	454	76	19	47	197	67	122	67
Ler-0	CS77020	10.87	47.98	Germany	Bayern	80	85	32	6507	220	-44	264	161	7	161	-6	965	123	54	32	358	166	358	172
Olympia-2	CS77144	21.62	37.63	Greece	Dytiki Ellada	176	94	37	5495	313	65	248	125	246	248	110	774	147	5	75	394	20	42	354
Sap-0	CS76596	141.35	43.06	Japan	Hokkaido	83	79	22	9427	259	-87	346	169	116	202	-38	1153	140	58	28	395	190	355	309
Tol-0	CS76614	-83.56	41.66	USA	Ohio	98	108	28	9782	297	-87	384	221	-18	221	-32	843	93	46	20	260	157	260	162
TRE-1	CS77385	4.10	48.86	France	Marne	101	93	37	5744	240	-10	250	173	63	173	26	632	68	39	14	180	129	180	155
Tri-0	CS77386	-6.01	37.38	Spain	Sevilla	186	121	39	6105	361	56	305	119	265	266	110	574	87	1	64	245	18	22	230
Ws-2	CS76631	30.00	52.30	Belarus	Gomel	67	87	25	9081	242	-100	342	179	-44	179	-53	624	87	30	34	241	99	241	111

Genotype	Stock number	Longitude	Latitude	Country	State/Province	Methylation contexts			Transposon superfamilies											
						mCG	mCHG	mCHH	Gypsy	Copia	LINE	SINE	En-Spm	Harbinger	HAT	Mariner	MuDR	Pogo	Tc1	Helitron
Abd-0	CS76429	-2.22	57.15	United Kingdom	Aberdeen	0.051	0.021	0.038	655.0	290.3	158.5	62.8	150.2	65.4	207.5	45.1	698.8	83.9	24.1	1786.5
Ang-0	CS76436	5.30	50.30	Belgium	Namur	0.029	0.008	0.013	761.9	305.7	174.5	52.5	168	58.9	186.4	31.9	602.2	58.2	16.4	1342.8
Col-0	CS76778	-92.30	38.30	USA	Missouri	0.043	0.02	0.034	587.5	310.5	163.5	65.5	116.5	67	247.7	70.6	814.8	136.2	32.1	2289.9
Dja-1	CS76473	73.62	42.59	Kyrgyzstan	Chui	0.043	0.017	0.028	756.0	385	164.9	52.1	155.7	51	170.5	31	595	54.3	12.8	1181.9
En-1	CS76841	8.50	50.00	Germany	Hessen	0.033	0.011	0.015												
Fei-0	CS76412	-8.54	40.92	Portugal	Aveiro	0.048	0.014	0.019												
Kar-1	CS76522	74.37	42.30	Kyrgyzstan	Chui	0.025	0.008	0.015												
Ler-0	CS77020	10.87	47.98	Germany	Bayern	0.025	0.007	0.012												
Olympia-2	CS77144	21.62	37.63	Greece	Dytiki Ellada	0.029	0.01	0.013												
Sap-0	CS76596	141.35	43.06	Japan	Hokkaido	0.027	0.007	0.013	617.5	304.6	155.1	57.4	145.8	73	244.2	65	776.7	115.6	31.3	2121.8
Tol-0	CS76614	-83.56	41.66	USA	Ohio	0.036	0.011	0.015	729.6	319	157.7	53.5	153.9	57.8	204.4	42.4	632.3	81.4	20.2	1548.6
TRE-1	CS77385	4.10	48.86	France	Marne	0.027	0.007	0.012												
Tri-0	CS77386	-6.01	37.38	Spain	Sevilla	0.026	0.008	0.015												
Ws-2	CS76631	30.00	52.30	Belarus	Gomel	0.044	0.017	0.013	774.8	372.4	177	55.3	162.1	52.3	171.2	32.3	588.8	58.6	15.5	1214.6

Supplementary Table 2 | The statistics results of linear mixed-effect models applied to phenotypes measured on the ancestor generation and each offspring generation of Test I. All statistical tests are two-sided.

		Flowering time			Plant height			Aboveground biomass			Rosett diameter			Fruit number		
	df1/df	*df2*	*F/Chi-sq*	*P*	*df2*	*F/Chi-sq*	*P*	*df2*	F/Chi-sq	*P*	*df2*	F/Chi-sq	*P*	*df2*	*F/Chi-sq*	*P*
Ancestor generation																
**Fixed effects**																
Genotype (G)	13	716.0	196.92	**<0.001**	728.8	102.94	**<0.001**	743.2	96.25	**<0.001**	552.0	82.80	**<0.001**	709.5	20.50	**<0.001**
Environment (E)	10	50.7	2.74	**0.009**	50.6	13.23	**<0.001**	51.7	12.96	**<0.001**	48.6	9.93	**<0.001**	50.9	20.90	**<0.001**
G x E	127	716.9	1.15	0.134	729.5	1.84	**<0.001**	743.4	3.52	**<0.001**	552.6	1.58	**<0.001**	710.0	1.64	**<0.001**
**Random effects**																
A.Layer	1	-	12.63	**<0.001**	-	1.46	0.226	-	5.95	**0.015**	-	8.46	**0.004**	-	13.22	**<0.001**
A.Row	1	-	0.00	1.000	-	1.64	0.200	-	71.42	**<0.001**	-	6.09	**0.014**	-	3.25	0.071
F1 generation																
**Fixed effects**																
F1.Layer	11	13.4	5.24	**0.003**	13.6	8.21	**<0.001**	13.5	5.39	**0.002**	13.3	10.67	**<0.001**	13.6	12.37	**<0.001**
Genotype (G)	13	727.8	177.82	**<0.001**	737.4	226.23	**<0.001**	734.5	138.87	**<0.001**	698.7	114.63	**<0.001**	736.8	14.28	**<0.001**
Ancestral Environment (A.E)	10	45.1	1.51	0.166	45.6	1.83	0.082	45.5	1.89	0.072	44.7	2.50	**0.018**	45.5	1.14	0.352
G x A.E	126	728.0	2.49	**<0.001**	738.2	1.96	**<0.001**	735.2	1.97	**<0.001**	698.8	1.58	**<0.001**	737.5	1.36	**0.009**
**Random effects**																
A.Layer	1	-	0.00	1.000	-	0.00	1.000	-	0.00	1.000	-	0.00	1.000	-	0.00	1.000
A.Row	1	-	0.00	1.000	-	0.00	1.000	-	0.13	0.072	-	0.00	1.000	-	1.21	0.271
F2 generation																
**Fixed effects**																
F2.Layer	11	13.6	5.60	**0.002**	14.3	5.55	**0.002**	13.5	20.15	**<0.001**	13.2	12.27	**<0.001**	13.4	16.34	**<0.001**
Genotype (G)	13	761.4	119.03	**<0.001**	751.4	255.63	**<0.001**	757.8	108.20	**<0.001**	737.7	118.04	**<0.001**	749.5	28.13	**<0.001**
Ancestral Environment (A.E)	10	45.9	1.05	0.421	46.2	1.51	0.167	45.8	2.31	**0.027**	45.2	1.44	0.194	45.6	1.19	0.326
G x A.E	126	761.5	2.06	**<0.001**	751.5	2.07	**<0.001**	757.9	1.74	**<0.001**	738.0	1.42	**0.003**	749.7	1.24	0.052
**Random effects**																
A.Layer	1	-	0.00	1.000	-	0.17	1.000	-	0.00	1.000	-	0.00	1.000	-	0.00	1.000
A.Row	1	-	0.00	1.000	-	0.00	1.000	-	0.00	1.000	-	0.00	1.000	-	0.02	0.892
F3 generation																
**Fixed effects**																
F3.Layer	11	13.6	16.76	**<0.001**	13.4	2.79	**0.039**	13.4	20.81	**<0.001**	13.5	8.43	**<0.001**	13.4	17.57	**<0.001**
Genotype (G)	13	756.1	178.65	**<0.001**	748.8	206.79	**<0.001**	736.9	132.11	**<0.001**	692.9	149.09	**<0.001**	749.6	18.05	**<0.001**
Ancestral Environment (A.E)	10	45.8	1.91	0.068	45.5	1.74	0.100	45.4	1.45	0.191	44.5	1.36	0.228	45.5	0.77	0.661
G x A.E	126	756.0	2.80	**<0.001**	748.8	1.92	**<0.001**	737.1	1.85	**<0.001**	692.5	1.53	**<0.001**	749.5	0.92	0.726
**Random effects**																
A.Layer	1	-	0.00	1.000	-	0.00	1.000	-	0.00	1.000	-	0.00	1.000	-	0.00	1.000
A.Row	1	-	0.00	1.000	-	0.00	1.000	-	0.00	1.000	-	1.45	0.228	-	0.02	0.878
F4 offspring generation																
**Fixed effects**																
F4.Layer	11	13.2	7.38	**<0.001**	13.8	6.29	**0.001**	13.0	18.30	**<0.001**	12.5	8.57	**<0.001**	13.9	21.57	**<0.001**
Genotype (G)	13	727.7	140.06	**<0.001**	707.3	123.55	**<0.001**	670.7	102.06	**<0.001**	605.9	56.88	**<0.001**	709.1	21.13	**<0.001**
Ancestral Environment (A.E)	10	45.3	1.98	0.058	45.5	1.58	0.143	43.9	1.96	0.062	42.6	0.83	0.600	45.5	0.34	0.964
G x A.E	126	727.7	2.81	**<0.001**	707.3	1.74	**<0.001**	670.4	2.09	**<0.001**	602.8	1.42	**0.004**	709.2	1.08	0.266
**Random effects**																
A.Layer	1	-	0.00	1.000	-	0.15	0.694	-	0.00	1.000	-	0.00	1.000	-	0.12	0.732
A.Row	1	-	0.37	0.545	-	0.00	1.000	-	0.00	1.000	-	0.00	1.000	-	0.00	1.000

Supplementary Table 3 | Statistical results comparing phenotypes (Test I) between different offspring generations derived from the ancestor environment of control. All statistical tests are two-sided.

	Flowering time				Plant height			Aboveground biomass			Rosett diameter			Fruit number		
	df1	*df2*	*F*	*P*	*df2*	*F*	*P*	*df2*	*F*	*P*	*df2*	*F*	*P*	*df2*	*F*	*P*
Genotype (G)	13	572.2	111.16	**<0.001**	569.8	191.66	**<0.001**	565.3	86.41	**<0.001**	533.7	71.56	**<0.001**	569.6	13.64	**<0.001**
Generation (F)	3	570.5	153.43	**<0.001**	568.4	37.91	**<0.001**	564.5	132.27	**<0.001**	532.8	161.77	**<0.001**	568.4	119.46	**<0.001**
G*F	39	570.6	6.40	**<0.001**	568.4	4.85	**<0.001**	564.4	3.46	**<0.001**	532.8	2.64	**<0.001**	568.4	1.16	0.238
A.layer	1	-	0.00	1.000	-	0.00	1.000	-	0.00	1.000	-	0.00	1.000	-	0.00	1.000
A.row	1	-	0.00	1.000	-	0.00	1.000	-	0.00	1.000	-	0.00	1.000	-	0.00	1.000

Supplementary Table 4 | The statistics results of linear mixed-effect models applied to all generations of Test I. Because generation (F) is nested in the factor layer in Test I, removing the layer effect also removes the generation effect. All statistical tests are two-sided.

	Flowering time				Plant height			Aboveground biomass			Rosett diameter			Fruit number		
	df1/df	*df2*	*F/Chi-sq*	*P*	*df2*	*F/Chi-sq*	*P*	*df2*	*F/Chi-sq*	*P*	*df2*	*F/Chi-sq*	*P*	*df2*	*F/Chi-sq*	*P*
**Fixed effects**																
Layer	47	240.7	8.04	**<0.001**	240.0	6.42	**<0.001**	239.0	16.93	**<0.001**	235.2	11.22	**<0.001**	239.2	17.79	**<0.001**
Genotype (G)	13	762.0	407.07	**<0.001**	761.9	468.74	**<0.001**	755.8	340.38	**<0.001**	740.6	330.16	**<0.001**	755.7	56.91	**<0.001**
Ancestral Environment (A.E)	10	45.7	2.22	**0.033**	45.7	2.34	**0.025**	45.5	3.32	**0.003**	44.6	2.78	**0.009**	45.5	0.94	0.509
G x A.E	126	762.9	4.18	**<0.001**	763.0	3.11	**<0.001**	758.7	3.23	**<0.001**	743.1	2.37	**<0.001**	757.9	1.31	**0.019**
G x F	39	2387.7	32.51	**<0.001**	2358.3	23.38	**<0.001**	2329.9	18.13	**<0.001**	2211.0	11.38	**<0.001**	2375.6	7.21	**<0.001**
A.E x F	30	2386.1	1.51	**0.038**	2357.3	1.25	0.161	2329.0	1.33	0.112	2200.0	1.36	0.094	2373.8	0.90	0.620
G x A.E x F	377	2390.7	1.86	**0.000**	2362.4	1.22	**0.005**	2336.7	1.28	**<0.001**	2216.9	1.15	**0.036**	2380.2	1.05	0.250
**Random effects**																
Line	1	-	63.42	**<0.001**	-	134.89	**<0.001**	-	42.04	**<0.001**	-	20.71	**<0.001**	-	3.05	0.081
A.Layer	1	-	0.00	1.000	-	0.00	1.000	-	0.00	1.000	-	0.00	1.000	-	0.00	1.000
A.Row	1	-	0.00	1.000	-	0.00	1.000	-	0.00	1.000	-	0.00	0.999	-	0.01	0.915

Supplementary Table 5 | The statistics results of linear mixed-effect models applied to phenotypes measured on the ancestor generation and each offspring generation of Test II. The statistics for F1 may be biased due to missing samples. All statistical tests are two-sided.

	Flowering time				Plant height			Aboveground biomass			Rosett diameter			Fruit number		
	df1/df	*df2*	*F/Chi-sq*	*P*	*df2*	*F/Chi-sq*	*P*	*df2*	*F/Chi-sq*	*P*	*df2*	*F/Chi-sq*	*P*	*df2*	*F/Chi-sq*	*P*
F1 generation																
**1st offspring generation**																
**Fixed effects**																
F1.Layer	15	175.9	2.40	**0.003**	172.3	3.74	**<0.001**	170.8	7.37	**<0.001**	156.7	3.97	**<0.001**	170.1	5.44	**<0.001**
Genotype (G)	9	160.7	22.29	**<0.001**	157.6	59.52	**<0.001**	156.5	17.39	**<0.001**	144.2	33.42	**<0.001**	156.0	8.05	**<0.001**
Ancestral Environment (A.E)	5	31.8	1.77	0.147	31.5	3.69	**0.010**	31.2	1.77	0.148	29.0	1.32	0.283	30.2	1.22	0.323
G x A.E	28	161.2	2.23	**0.001**	157.7	1.28	0.173	156.6	1.01	0.453	143.6	1.61	**0.038**	156.0	1.33	0.141
**Random effects**																
A.Layer	1	-	0.07	0.789	-	0.00	1.000	-	0.13	0.720	-	4.22	**0.040**	-	3.60	0.058
A.Row	1	-	0.00	1.000	-	0.11	0.738	-	0.00	0.989	-	0.00	1.000	-	0.00	1.000
F2 generation																
**Fixed effects**																
F2.Layer	15	243.1	2.60	**0.001**	241.9	4.44	**<0.001**	224.5	8.30	**<0.001**	218.4	4.70	**<0.001**	241.9	5.79	**<0.001**
Genotype (G)	9	222.6	17.01	**<0.001**	223.9	44.04	**<0.001**	208.7	19.27	**<0.001**	202.7	19.41	**<0.001**	223.9	8.39	**<0.001**
Ancestral Environment (A.E)	5	26.1	0.67	0.647	26.6	1.50	0.222	26.0	2.30	0.074	25.5	1.16	0.356	26.6	1.21	0.332
G x A.E	41	222.9	1.89	**0.002**	223.6	2.35	**<0.001**	208.4	1.58	**0.021**	201.6	1.93	**0.002**	223.6	1.17	0.233
**Random effects**																
A.Layer	1	-	1.28	0.259	-	0.00	0.991	-	0.00	1.000	-	0.00	1.000	-	0.00	1.000
A.Row	1	-	0.19	0.662	-	0.00	1.000	-	0.00	1.000	-	0.01	0.931	-	0.00	1.000
F3 generation																
**Fixed effects**																
F3.Layer	15	248.5	3.71	**<0.001**	249.1	2.99	**<0.001**	228.0	5.92	**<0.001**	243.9	3.01	**<0.001**	248.2	5.83	**<0.001**
Genotype (G)	9	230.5	40.59	**<0.001**	230.8	63.62	**<0.001**	213.7	25.24	**<0.001**	226.7	36.83	**<0.001**	229.9	14.49	**<0.001**
Ancestral Environment (A.E)	5	25.8	3.44	**0.016**	26.4	1.62	0.189	25.2	1.66	0.181	26.2	2.74	**0.041**	25.8	1.93	0.124
G x A.E	41	229.8	2.90	**<0.001**	229.8	1.79	**0.004**	212.3	2.27	**<0.001**	225.5	2.36	**<0.001**	229.0	1.24	0.166
**Random effects**																
A.Layer	1	-	3.51	0.061	-	0.03	0.862	-	0.00	1.000	-	0.03	0.872	-	3.68	0.055
A.Row	1	-	0.00	1.000	-	0.00	1.000	-	0.00	1.000	-	0.00	1.000	-	0.00	1.000
F4 generation																
**Fixed effects**																
F4.Layer	15	242.1	3.44	**<0.001**	240.4	2.58	**0.001**	233.8	9.13	**<0.001**	225.8	5.14	**<0.001**	239.8	3.72	**<0.001**
Genotype (G)	9	226.5	48.58	**<0.001**	222.5	58.28	**<0.001**	215.2	31.66	**<0.001**	213.9	42.06	**<0.001**	224.9	15.88	**<0.001**
Ancestral Environment (A.E)	5	26.7	1.58	0.200	26.8	0.90	0.495	25.6	2.52	0.055	26.0	1.92	0.126	26.7	0.34	0.883
G x A.E	41	226.5	3.12	**<0.001**	222.5	2.14	**<0.001**	215.2	2.79	**<0.001**	213.0	1.32	0.106	224.9	1.56	**0.023**
**Random effects**																
A.Layer	1	-	0.00	1.000	-	0.00	1.000	-	0.54	0.463	-	0.00	1.000	-	0.00	1.000
A.Row	1	-	0.00	1.000	-	0.87	0.350	-	0.00	1.000	-	0.00	1.000	-	0.00	1.000

Supplementary Table 6 | Statistical results comparing phenotypes (Test II) between different offspring generations derived from the ancestor environment of control. All statistical tests are two-sided.

	Flowering time				Plant height			Aboveground biomass			Rosett diameter			Fruit number		
	df1	*df2*	*F*	*P*	*df2*	*F*	*P*	*df2*	*F*	*P*	*df2*	*F*	*P*	*df2*	*F*	*P*
Genotype (G)	9	172.8	22.56	**<0.001**	165.1	46.08	**<0.001**	161.4	14.49	**<0.001**	168.6	16.24	**<0.001**	165.1	8.65	**<0.001**
Generation (F)	3	173.7	28.45	**<0.001**	175.1	2.18	0.093	165.6	9.88	**<0.001**	167.1	3.37	**0.020**	175.1	1.36	0.256
G*F	27	174.2	3.31	**<0.001**	175.5	1.25	0.200	166.0	3.32	**<0.001**	167.7	2.16	**0.002**	175.5	2.07	**0.003**
A.layer	1	-	0.00	1.000	-	0.00	1.000	-	0.00	1.000	-	0.00	1.000	-	0.00	1.000
A.row	1	-	0.00	1.000	-	0.00	1.000	-	0.00	1.000	-	0.00	1.000	-	0.00	1.000

Supplementary Table 7 | The statistics results of linear mixed-effect models applied to all generations of Test II. All statistical tests are two-sided.

	Flowering time				Plant height			Aboveground biomass			Rosett diameter			Fruit number		
	df1/df	*df2*	*F/Chi-sq*	*P*	*df2*	*F/Chi-sq*	*P*	*df2*	*F/Chi-sq*	*P*	*df2*	*F/Chi-sq*	*P*	*df2*	*F/Chi-sq*	*P*
**Fixed effects**																
Layer	15	972.7	8.45	**<0.001**	947.1	10.76	**<0.001**	907.4	28.03	**<0.001**	902.7	14.22	**<0.001**	961.7	16.90	**<0.001**
Genotype (G)	9	245.1	99.32	**<0.001**	246.7	160.84	**<0.001**	240.9	69.12	**<0.001**	241.9	99.77	**<0.001**	245.2	33.97	**<0.001**
Ancestral Environment (A	5	27.0	2.83	**0.035**	27.7	0.62	0.686	27.2	1.40	0.255	26.8	2.26	0.077	27.2	0.39	0.849
Generation(F)	3	727.5	160.09	**<0.001**	717.8	6.50	**<0.001**	685.5	90.78	**<0.001**	680.5	13.51	**<0.001**	721.1	2.58	0.053
G x A.E	41	255.4	5.63	**<0.001**	254.2	3.91	**<0.001**	249.9	3.30	**<0.001**	248.7	3.39	**<0.001**	254.2	1.38	0.073
G x F	27	728.8	9.14	**<0.001**	719.8	4.42	**<0.001**	687.2	5.05	**<0.001**	686.1	3.82	**<0.001**	723.0	3.05	**<0.001**
A.E x F	15	727.8	1.72	**0.043**	718.6	1.67	0.052	686.1	2.25	**0.004**	680.5	1.72	**0.043**	721.4	1.65	0.057
G x A.E x F	110	730.7	1.43	**0.004**	720.8	0.96	0.604	689.3	1.53	**<0.001**	685.8	1.08	0.287	723.9	1.28	**0.038**
**Random effects**																
Line	1	-	2.24	0.134	-	14.48	**<0.001**	-	8.53	**0.003**	-	6.48	**0.011**	-	4.52	**0.034**
A.Layer	1	-	1.65	0.198	-	0.00	1.000	-	0.00	1.000	-	0.11	0.739	-	0.62	0.430
A.Row	1	-	0.00	1.000	-	0.00	1.000	-	0.00	1.000	-	0.01	0.910	-	0.00	1.000
