# Supplementary material for: Environment-induced heritable variations are common in Arabidopsis thaliana
Source: Nat Commun. 2024 May 30;15:4615. doi: 10.1038/s41467-024-49024-3 (PMC11139905; doi:10.1038/s41467-024-49024-3)
Supplement: Supplementary file 3 — Reporting Summary [file 41467_2024_49024_MOESM3_ESM.pdf]

nature portfolio

Corresponding author(s): Yuan-Ye Zhang

Last updated by author(s): May 4, 2024

Reporting Summary

Nature Portfolio wishes to improve the reproducibility of the work that we publish. This form provides structure for consistency and transparency in reporting. For further information on Nature Portfolio policies, see our Editorial Policies and the Editorial Policy Checklist.

Statistics

For all statistical analyses, confirm that the following items are present in the figure legend, table legend, main text, or Methods section.

n/a	Confirmed	
☐	☒	The exact sample size (n) for each experimental group/condition, given as a discrete number and unit of measurement
☐	☒	A statement on whether measurements were taken from distinct samples or whether the same sample was measured repeatedly
☐	☒	The statistical test(s) used AND whether they are one- or two-sided<br>*Only common tests should be described solely by name; describe more complex techniques in the Methods section.*
☐	☒	A description of all covariates tested
☐	☒	A description of any assumptions or corrections, such as tests of normality and adjustment for multiple comparisons
☐	☒	A full description of the statistical parameters including central tendency (e.g. means) or other basic estimates (e.g. regression coefficient) AND variation (e.g. standard deviation) or associated estimates of uncertainty (e.g. confidence intervals)
☐	☒	For null hypothesis testing, the test statistic (e.g. F, t, r) with confidence intervals, effect sizes, degrees of freedom and P value noted<br>*Give P values as exact values whenever suitable.*
☒	☐	For Bayesian analysis, information on the choice of priors and Markov chain Monte Carlo settings
☐	☒	For hierarchical and complex designs, identification of the appropriate level for tests and full reporting of outcomes
☐	☒	Estimates of effect sizes (e.g. Cohen's d, Pearson's r), indicating how they were calculated

Our web collection on statistics for biologists contains articles on many of the points above.

Software and code

Policy information about availability of computer code

Data collection	No software was used to collect data, and the phenotype and RNA-seq data were obtained from our experiments. (see our declaration of data availability for details).
Data analysis	The custom pipelines and scripts were deposited at GitHub (https://github.com/Xiaohe-Lin/Heritable_variation). All softwares used in the present study are publicly available and the specifying versions are listed as follows: Trimmomatic v0.36, bwa-meth v0.2.5, SAMtools v1.7, Picard v2.18.10, MethylDackel v0.6.1, STAR v2.7.9, gffread v.0.12.7, featureCounts v1.22.2, R v3.6.2 and R packages (lme4 v1.1.30, lmerTest v3.1.3, scatterpie v0.1.8, car v3.1.1, multcomp v1.4.20, raster v3.6.20, ggplot2 v3.4.2, DESeq2 v1.36.0, clusterProfiler v4.4.4, pheatmap v1.0.12, and GenomicRanges v1.42.0).

For manuscripts utilizing custom algorithms or software that are central to the research but not yet described in published literature, software must be made available to editors and reviewers. We strongly encourage code deposition in a community repository (e.g. GitHub). See the Nature Portfolio guidelines for submitting code & software for further information.

Data

Policy information about availability of data

All manuscripts must include a data availability statement. This statement should provide the following information, where applicable:

- Accession codes, unique identifiers, or web links for publicly available datasets
- A description of any restrictions on data availability
- For clinical datasets or third party data, please ensure that the statement adheres to our policy

The phenotypic data of the ancestral generation, Test I and Test II are available on GitHub (https://github.com/Xiaohe-Lin/Heritable_variation). Raw RNA-seq data are available from the NCBI SRA database with accession ID PRJNA997595.The geographical and climatic data sourced from WorldClim (https://worldclim.org), and information of DNA methylation and transposon abundance were obtained from published literature (Kawakatsu. et al. Cell, 2016, 166:492-505; Quadrana. et al. eLife, 2016, 5:e15716). The TAIR 10 reference genome of Arabidopsis thaliana and annotations are obtained from Arabidopsis Information Resource (https://www.arabidopsis.org).

Research involving human participants, their data, or biological material

Policy information about studies with human participants or human data. See also policy information about sex, gender (identity/presentation), and sexual orientation and race, ethnicity and racism.

Reporting on sex and gender	N/A
Reporting on race, ethnicity, or other socially relevant groupings	N/A
Population characteristics	N/A
Recruitment	N/A
Ethics oversight	N/A

Note that full information on the approval of the study protocol must also be provided in the manuscript.

Field-specific reporting

Please select the one below that is the best fit for your research. If you are not sure, read the appropriate sections before making your selection.

☐ Life sciences ☐ Behavioural & social sciences ☒ Ecological, evolutionary & environmental sciences

For a reference copy of the document with all sections, see nature.com/documents/nr-reporting-summary-flat.pdf

Ecological, evolutionary & environmental sciences study design

All studies must disclose on these points even when the disclosure is negative.

Study description	Parental or ancestral environments can induce heritable phenotypic changes, but whether such environment-induced heritable changes are a common phenomenon remains unexplored. Here, we subjected 14 genotypes of Arabidopsis thaliana to the control and different environmental treatments and observed phenotypic and genome-wide gene expression changes over successive generations. In this study, the ancestral environments included 14 genotypes, and environments of the control and ten treatments, each treatment included six replicate plants per genotype, and the control included 12 plants per genotype, resulting in 1008 plants grown or lines established in the ancestral generation. We then planted one descendant per line, following the single-seed descendant approach, and planted 1008 lines generation-by-generation in the control environment for four generations (F1-F4). The growth room contained four shelves, each with four layers. In each layer, we organized the plants into six rows and 14 columns, and this organization allowed one replicate per treatment/genotype to be planted in two layers. For one replicate of the ancestral generation, treatments were randomly assigned to rows in two layers, and genotypes were distributed randomly among columns in a row. For the offspring of this replicate, we assigned them to two random layers and distributed the plants randomly among different positions. To eliminate the potential influence of growth conditions that varied between generations, we planted all four offspring generations together in the control environment including a subset of ten genotypes and six different environments, resulting in 360 plants per offspring generation and 1440 plants for four offspring generations. We adopted a completely random design, assigning the 1440 plants randomly into positions among the 16 layers, with six rows and 15 columns each. We measured phenotypes (flowering time, plant height, aboveground biomass, rosette leaf diameter and fruit number) from the ancestral and offspring generations in Test I and Test II, and collected RNA-seq data from the offspring generations in Test II. To assess the effects of the environmental treatment and genotype on the ancestral generation, we applied a linear mixed-effect model to each phenotype, including genotype (G), environment (E), and their interaction (G × E) as fixed effects and the layer (A.Layer) and row (A.Row) as random effects. To assess the effects of ancestral treatment and genotype on offspring generations, we applied a linear mixed-effect model to each phenotype, including offspring layer (O. Layer), genotype (G), ancestral environment (A.E), and their interaction (G × A.E) as fixed effects and ancestor layer (A. Layer) and row (A. Row) as random effects. Furthermore, we transformed the quantitative

	estimates of effect sizes into qualitative assessments (occurrences) to investigate the prevalence of these changes across generations. To investigate the predictability environment-induced phenotypic changes, we applied generalized linear mixed-effect models to the qualitative occurrence data. To explore which factors could predict the genotypic variation in the occurrence of transgenerational effects, we related the occurrence probability of genotypes to climates at the origin sites, the DNA methylation level, and the number of transposons in the genomes. To reveal the reproducibility of environment-induced phenotypic changes, we calculated the Pearson correlation between generations and Tests. Moreover, using RNA-seq data, we identified the number of differentially expressed genes (DEGs) for each genotype, ancestral environment treatment and offspring generation and extracted DEGs differentially expressed in at least two generations (heritable DEGs) to explore the factors that explained the number of DEGs. To investigate the function of DEGs, we focused on heritable DEGs and conducted KEGG and Gene Ontology (GO) enrichment analysis and summarized their overlapping patterns between genotypes and environments. To investigate whether the heritable phenotypic changes were caused by heritable gene expression changes, we focused on flowering time and identified 79 flowering time-related genes from the literature.
Research sample	We ordered 14 A. thaliana genotypes from the Arabidopsis Biological Resource Centre ABRC, including Abd-0 (from Aberdeen, UK), Ang-0 (Namur, Belgium), Col-0 (Columbia, US), Dja-1 (Chui, Kyrgyzstan), En-1 (Frankfurt, Germany), Fei-0 (Aveiro, Portugal), Kar-1 (Suusamyr, Kyrgyzstan), Ler-0 (Munich, Germany), Olympia-2 (Olympia, Greece), Sap-0 (Hokkaido, Japan), Tol-0 (Ohio, USA), TRE-1 (Marne, France), Tri-0 (Sevilla, Spain) and Ws-2 (Belarus, Belarus). The stock IDs are shown in Supplementary Table 1. The geographic locations (longitudes/latitudes) where the seeds were originally collected are shown in Supplementary Fig. 1a and Supplementary Table 1.
Sampling strategy	For the phenotypic data, we included six biological replicates, which are commonly seen in transgenerational effect studies (Dooren et al 2020 Journal of Experimental Botany 22;71(12):3588-3602.) and meet the requirements for statistical analysis of linear mixed models and generalized linear models. For the transcriptome data, we included three biological replicate samples (6G per sample, approximately 50x), which is sufficient to provide accurate transcriptome differential analysis results (Robles et al. 2021 BMC Genomics 2012, 17; 13:484).
Data collection	The collection of phenotypic data was carried out by Junje Yin, Jing Yao, Yifan Yao and Xiaohe Lin. Experiments of the RNA extraction were conducted by Xiaohe Lin. Transcriptomic raw reads were collected using Illumina platforms, through the sequencing service of Novogene.
Timing and spatial scale	Data collection in this study started from March 2018 and finished in May 2022. From March 2018 to December 2022, we establish the ancestral (March-August 2018), F1 (May-October 2019), F2 (May-October 2019), F3 (July 2020 to January 2021), F4 (November 2020 to March 2021) generations of Test I and Test II (August 2021 to January 2022) to collect five phenotypic traits (Supplementary Fig. 1d). From November 2021 to May 2022, we collected leaf tissues used for transcriptome analysis.
Data exclusions	No data were excluded.
Reproducibility	All attempts to repeat the experiment were successful.
Randomization	The growth room contained four shelves, each with four layers (Supplementary Fig. 1b). For test I, in each layer, we organized the plants into six rows and 14 columns, and this organization allowed one replicate per treatment/genotype to be planted in two layers. For one replicate of the ancestral generation, treatments were randomly assigned to rows in two layers, and genotypes were distributed randomly among columns in a row (Supplementary Fig. 1c). For the offspring of this replicate, we assigned them to two random layers and distributed the plants randomly among different positions (Supplementary Fig. 1c). All randomization was conducted using the "RAND" function in Excel. For Test II, we also adopted a completely random design, assigning the 1440 plants randomly into positions among the 16 layers, with six rows and 15 columns each.
Blinding	All the investigators were blinded to group allocation during data collection.

Did the study involve field work? ☐ Yes ☒ No

Reporting for specific materials, systems and methods

We require information from authors about some types of materials, experimental systems and methods used in many studies. Here, indicate whether each material, system or method listed is relevant to your study. If you are not sure if a list item applies to your research, read the appropriate section before selecting a response.

Materials & experimental systems

n/a	Involved in the study	
☒	☐	Antibodies
☒	☐	Eukaryotic cell lines
☒	☐	Palaeontology and archaeology
☒	☐	Animals and other organisms
☒	☐	Clinical data
☒	☐	Dual use research of concern
☐	☒	Plants

Methods

n/a	Involved in the study	
☒	☐	ChIP-seq
☒	☐	Flow cytometry
☒	☐	MRI-based neuroimaging

Dual use research of concern

Policy information about dual use research of concern

Hazards

Could the accidental, deliberate or reckless misuse of agents or technologies generated in the work, or the application of information presented in the manuscript, pose a threat to:

No	Yes	
☒	☐	Public health
☒	☐	National security
☒	☐	Crops and/or livestock
☒	☐	Ecosystems
☒	☐	Any other significant area

Experiments of concern

Does the work involve any of these experiments of concern:

No	Yes	
☒	☐	Demonstrate how to render a vaccine ineffective
☒	☐	Confer resistance to therapeutically useful antibiotics or antiviral agents
☒	☐	Enhance the virulence of a pathogen or render a nonpathogen virulent
☒	☐	Increase transmissibility of a pathogen
☒	☐	Alter the host range of a pathogen
☒	☐	Enable evasion of diagnostic/detection modalities
☒	☐	Enable the weaponization of a biological agent or toxin
☒	☐	Any other potentially harmful combination of experiments and agents

Plants

Seed stocks	Seeds collected from different geographic sites, known as natural accessions or ecotypes, are preserved at the Arabidopsis Biological Resource Centre (ABRC).
Novel plant genotypes	N/A
Authentication	N/A
