# Supplementary material for: Environment-induced heritable variations are common in Arabidopsis thaliana
Source: Nat Commun. 2024 May 30;15:4615. doi: 10.1038/s41467-024-49024-3 (PMC11139905; doi:10.1038/s41467-024-49024-3)
Supplement: Supplementary file 4 — Description of Additional Supplementary Files [file 41467_2024_49024_MOESM4_ESM.pdf]

Description of Additional Supplementary Files

File Name: Supplementary Data 1

Description: Transcriptomic sequencing information and mapping results for each sample.

File Name: Supplementary Data 2

Description: Significantly enriched GO terms of heritable DEGs for each genotype and treatment after simplification.

File Name: Supplementary Data 3

Description: Significantly enriched KEGG pathways of heritable DEGs for each genotype and treatment.

File Name: Supplementary Data 4

Description: Differential expression patterns of flowering time related genes.
